# The Styloid Muscles and Anæsthetics

**Published:** 1874-08

**Authors:** G. W. Copeland

**Affiliations:** BOSTON.


### ARTICLE VII.

### *The Styloid Muscles and Anæsthetics.*

BY G. W. COPELAND, M. D., BOSTON.

Anæsthetics have of late received a large share of consideration from the medical profession; yet so long as deaths occur from their use, it is evident that there is a fault, either in the drug or in the manner in which it is administered. Chloroform has been disposed of for the present, on the ground that it contains a poisonous ingredient liable, under any circumstances to produce death. Ether is more fortunate, for it has escaped censure.

Every medical man is familiar with the fact that, at a certain stage of anæsthesia, the breathing frequently becomes irregular and obstructed by what is termed "the tongue falling back in the throat." Let us look for one moment at the way in which the drug is invariably administered.

The patient, whether sitting in a chair, or lying in the recumbent posture, has his head tilted back, and is requested to open his mouth and "breathe hard." Now see what follows. So long as voluntary efforts on the part of the patient continue, all goes on well; but when unconsciousness takes place, the breathing then involuntary, becomes impeded and oppressed. Several devices are immediately resorted to, such as drawing the tongue out with a tenaculum, a pair of forceps, or, as I recently saw in one of our hospitals, by a ligature passed through it and held by the fingers of an assistant. These procedures generally help, but seldom wholly relieve the difficulty, so that the unconscious effort of the patient struggling for breath continues a source of anxiety to the assistants, and often to the surgeon.

Having occasion to use ether quite frequently, and many times with no other help than the friends of the patient, I have at times been greatly troubled and embarrassed by the occurrence of this state of affairs. Failing, one day, to relieve the trouble by the usual plan of drawing the tongue out, I tilted the head forwards, closing the mouth, and established free and regular respirations through the natural channel, the nose. Since that time, I have repeatedly relieved the difficulty in this simple way.

Let us see what happens when the head is tilted back in the usual position. The styloid muscles are put on the stretch. The stylo glossi draw the tongue backwards, the stylo-hyoidei draw the os hyoides upwards, and the stylo-pharyngei raise the pharynx and thyroid cartilage upwards, all thus uniting to close the epiglottis. Pulling out the tongue will partially overcome the action of the stylo glossi, while the other muscles still maintain their action.

Now see what takes place when the head is tilted forwards. The styloid muscles are all relaxed, the tongue falls forward in the mouth, and the larynx falls into its proper place, thus leaving the epiglottis free, and the glottis unobstructed.

Here, then, I claim, is the secret of preventing or relieving this trouble with the breathing. So soon as the patient becomes in the least unconscious, tilt the head forwards, and make him breathe through the nose, which certainly was made for that express purpose. It seems nothing more than fair that we should give our patient, when unconscious, the benefit of having his head in a natural position, or at least in the position which he maintains when conscious.—*Boston Medical and Surgical Journal.*

